# Acid-Sensing Ion Channel 1 Deficiency Aggravates Cuprizone-Induced Demyelination, Axonal Damage and Microgliosis in Mice

**DOI:** 10.3390/ijms27219758

**Published:** 2026-10-30

**Authors:** Ahmi Öz, Dominik Wiemuth, Stefan Gründer, Tim Clarner, Natalie Tillmann

**Affiliations:** 1 Anatomy and Cell Biology, University Hospital Bonn, University of Bonn, 53127 Bonn, Germany; timclarner@uni-bonn.de (T.C.); natalie.tillmann@uni-bonn.de (N.T.); 2 Department of Biophysics, Faculty of Medicine, Süleyman Demirel University, Isparta 32260, Türkiye; 3 Institute of Physiology, RWTH Aachen University, 52074 Aachen, Germany; dwiemuth@ukaachen.de (D.W.); sgruender@ukaachen.de (S.G.)

**Keywords:** ASIC1, oligodendrocytes, multiple sclerosis, cuprizone, myelin

## Abstract

White matter degeneration in multiple sclerosis (MS) is characterized by oligodendrocyte dysfunction and subsequent demyelination. To determine whether acid-sensing ion channel subtype 1 (ASIC1) contributes to oligodendrocyte-associated pathology under non-autoimmune conditions, the cuprizone model was used in wild-type (WT) and ASIC1-deficient (KO) mice. Axonal degeneration, microgliosis (IBA1), demyelination, and oligodendrocyte abundance were assessed in the medial Corpus Callosum. Cuprizone increased IBA1 positivity, axonal damage, and demyelination, and these effects appeared more pronounced in ASIC1 KO animals than in WT animals. ASIC1 deficiency was associated with increased APP^+^ axonal spheroid formation and reduced myelination scores. However, oligodendrocyte marker analyses did not indicate an additional reduction in oligodendrocyte survival after 3 weeks. Overall, these data support a role of ASIC1 in preserving myelin integrity, consistent with myelin maintenance rather than direct regulation of oligodendrocyte survival.

## 1. Introduction

Multiple sclerosis (MS) is a chronic autoimmune disease of the central nervous system (CNS) characterized by neuroinflammation, demyelination, and progressive neurodegeneration. In addition to inflammatory demyelinating lesions, MS pathology is associated with neuroaxonal injury, reactive gliosis, and tissue acidosis within active lesions [1,2,3]. Mechanistic studies have demonstrated that adaptive immune responses, particularly those mediated by autoreactive T lymphocytes, contribute substantially to immune attacks against myelin-associated structures, thereby impairing neuronal conduction and promoting progressive neurological dysfunction over time [4].

Despite the central role of immune-mediated pathology, increasing evidence suggests that primary cytodegenerative mechanisms also contribute to lesion formation and disease progression in MS. In particular, the molecular mechanisms underlying the primary degeneration of mature oligodendrocytes remain incompletely understood [5]. Biochemical alterations associated with MS lesions include oxidative stress mediated by reactive oxygen and nitrogen species, dysregulated ionic homeostasis involving disturbances in intracellular Ca^2+^ and Na^+^ levels, and mitochondrial dysfunction [6,7,8]. In addition, neuronal and oligodendroglial cell death has been linked to ion-channel-mediated dysregulation of cellular excitability and pathological ionic fluxes [9,10,11].

Oligodendrocytes are essential glial cells of the CNS that not only generate myelin sheaths but also provide metabolic and structural support to axons and contribute to the maintenance of axonal integrity [12,13]. Consequently, oligodendrocyte loss is thought to contribute directly to subsequent axonal degeneration and long-term neurological disability. Preventing oligodendrocyte degeneration and promoting efficient remyelination therefore represent major therapeutic goals in experimental MS research. Successful remyelination critically depends on the generation, differentiation, and maturation of oligodendrocyte precursor cells into myelinating oligodendrocytes [14]. However, these regenerative processes may be impaired within inflammatory lesions, as oligodendrocytes are particularly vulnerable to oxidative stress and metabolic disturbances. Moreover, their relatively limited antioxidant defenses and the lipid-rich composition of myelin further increase their susceptibility to cellular stress, emphasizing the importance of a supportive microenvironment for oligodendrocyte survival and function [15].

Acid-sensing ion channels (ASICs) belong to the epithelial sodium channel/degenerin (ENaC/DEG) superfamily and comprise six isoforms encoded by four genes in humans [16,17]. ASICs function as extracellular proton sensors and have been implicated in a variety of physiological and pathological processes within the nervous system [18,19]. Among these channels, acid-sensing ion channel 1 (ASIC1) is permeable to Na^+^ and, to a lesser extent, Ca^2+^ [20,21], and is expressed in multiple neural cell types, including oligodendrocytes [22,23,24]. In contrast to many other ligand-gated ion channels, ASIC1 is directly activated by extracellular acidosis, typically at pH values below approximately 6.8, with protons acting as the endogenous agonist [20,25].

Intracellular Ca^2+^ is a critical regulator of both physiological signaling pathways and pathological cell death mechanisms. Under conditions of Ca^2+^ overload, excessive cation influx through Ca^2+^-permeable channels can induce mitochondrial dysfunction, promote the formation of reactive oxygen species (ROS), and activate apoptotic signaling cascades [26]. Consistent with this concept, ASIC1-mediated Ca^2+^ influx has been implicated in neuronal injury and reduced cell viability, and several experimental approaches have therefore aimed to inhibit ASIC1 activity in order to attenuate neurodegenerative processes [27]. Despite these findings, the contribution of ASIC1 to oligodendrocyte degeneration and demyelinating pathology in MS remains insufficiently understood. Tissue acidosis is a characteristic feature of inflammatory CNS lesions and may arise from mitochondrial dysfunction, hypoxia-like metabolic alterations, and inflammatory cellular activity within active demyelinating plaques [3,6]. As proton-gated ion channels, ASICs are therefore well positioned to translate extracellular metabolic stress into intracellular signaling events. Previous studies have linked ASIC1 activation to neuronal injury and neurodegeneration in several CNS disorders, including ischemia and inflammatory diseases of the CNS [18,19,27]. However, comparatively little is known about the role of ASIC1 in oligodendrocyte pathology and demyelinating conditions.

In the present study, we investigated whether ASIC1 contributes to demyelination, gliosis, oligodendrocyte loss, and axonal damage in vivo using the toxic cuprizone model. To this end, cuprizone-induced pathology was analyzed in wild-type (WT) and ASIC1-deficient (KO) mice.

## 2. Results

### 2.1. ASIC1 Deficiency Increases IBA1-Positive Area in the Corpus Callosum of Cuprizone-Intoxicated Animals

**Figure 1:**
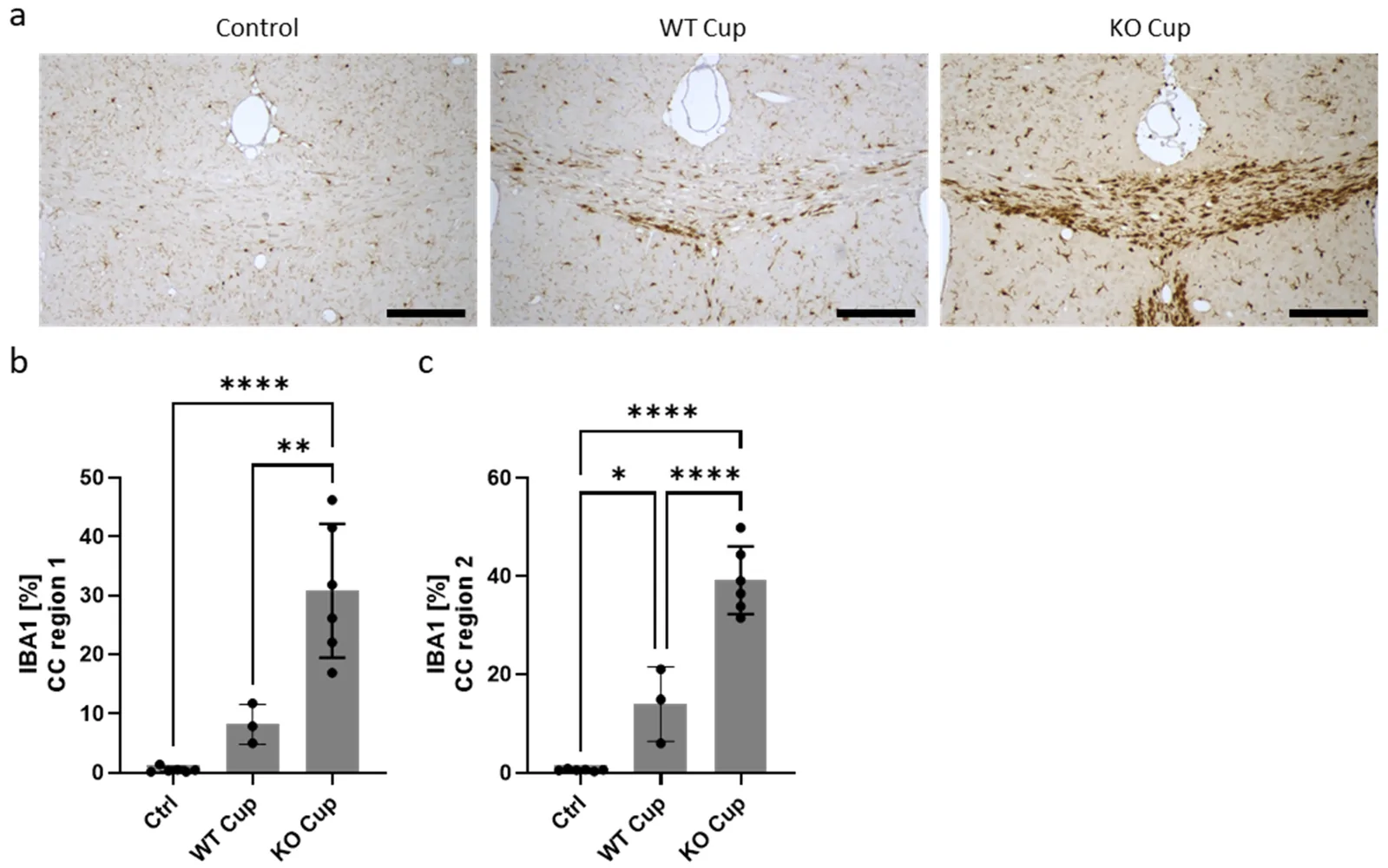
IBA1-positive area in the Corpus Callosum (CC) after 3 weeks of cuprizone intoxication. (**a**) Representative images of IBA1 staining in the CC of region 1. Scale bar 200 µm. (**b**) Densitometric analysis of IBA1^+^ area in % of the CC in region 1; results for region 2 are shown in (**c**). *n* = 6 (Ctrl, KO Cup), *n* = 3 (WT Cup). Mean ± SD. ANOVA with Tukey’s multiple comparison. * *p* ≤ 0.05; ** *p* ≤ 0.01; **** *p* ≤ 0.0001.

The effect of ASIC1 channel deficiency on microglia activation/microgliosis and/or macrophage infiltration in the brain of Cup-intoxicated animals was investigated by quantification of ionized calcium-binding adaptor molecule 1 (IBA1) staining in regions R1 and R2 of the Corpus Callosum (CC) in coronal sections. For densitometric analysis of IBA1 immunoreactivity, the medial part of the CC was chosen as the region of interest (ROI) in each slide. Representative images taken from R1 are given in Figure 1a. Densitometric quantifications are shown as bar graphs in Figure 1b,c for R1 and R2, respectively. Note that Cup intoxication increased the IBA1-positive area in the medial CC of mice. In R1, the IBA1-positive area in the ROI was 0.53 ± 0.44% in the Ctrl group, 8.23 ± 3.37% in WT Cup mice, and 30.78 ± 11.36% in KO Cup mice. In R2, 0.59 ± 0.18% of the ROI was IBA1-positive in the Ctrl group, compared with 13.99 ± 7.54% in WT Cup mice and 39.17 ± 6.88% in KO Cup mice.

### 2.2. Cuprizone Feeding Induced Increased Axonal Damage in the Corpus Callosum of ASIC1 KO Animals

**Figure 2:**
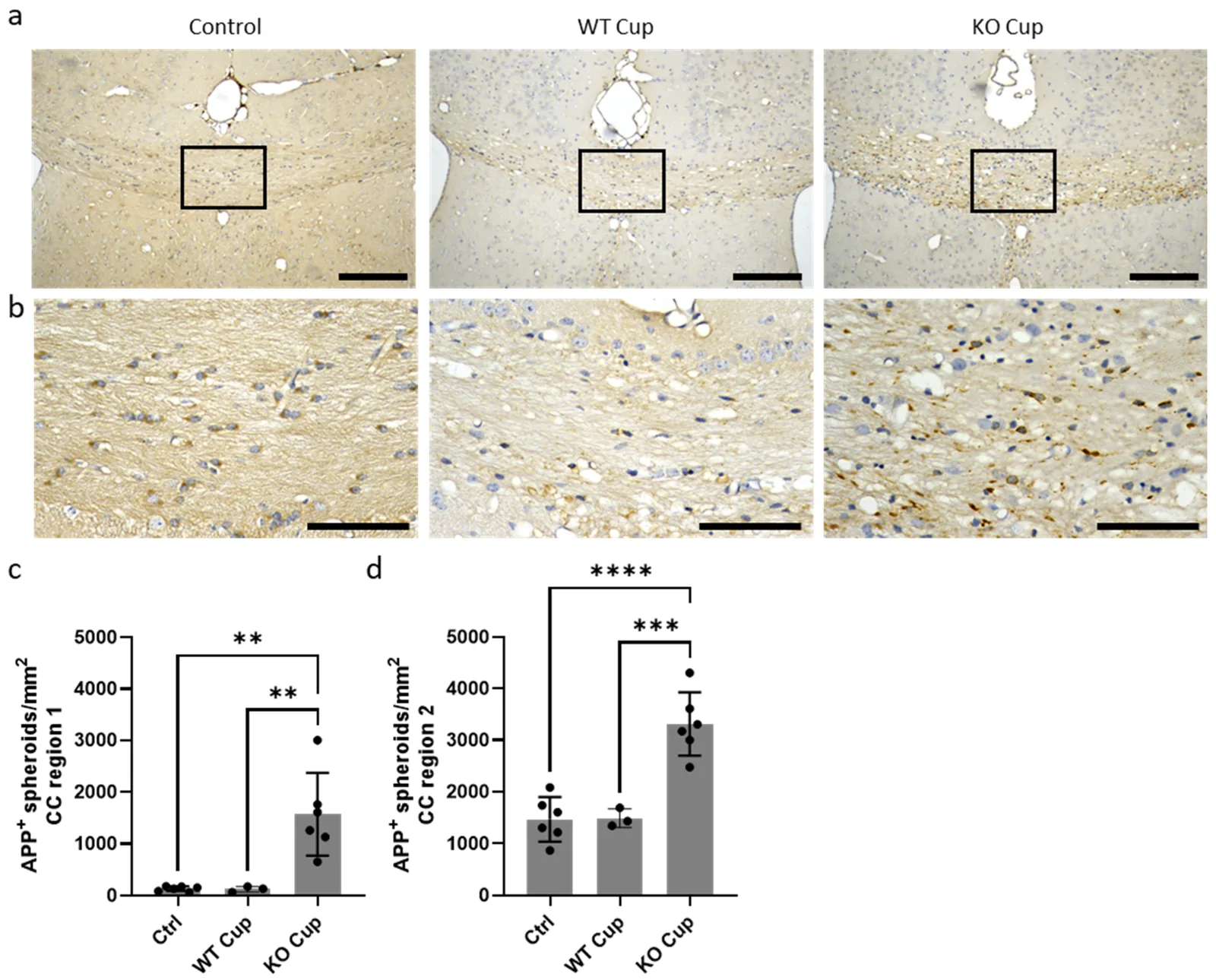
APP-positive spheroids in the Corpus Callosum (CC) after 3 weeks of cuprizone intoxication. (**a**) Representative images of APP staining in the CC of region 1. Scale bar 200 µm. (**b**) Higher magnification of the area outlined by the rectangle in (**a**), shown because APP^+^ spheroids are not resolvable at the magnification of (**a**). The ROI used for quantification is shown in Figure S3. Scale bar 50 µm. (**c**) Analysis of APP^+^ spheroids/mm^2^ in the CC of region 1; results for region 2 are shown in (**d**). *n* = 6 (Ctrl, KO Cup), *n* = 3 (WT Cup). Mean ± SD. ANOVA with Tukey’s multiple comparison. ** *p* ≤ 0.01; *** *p* ≤ 0.001; **** *p* ≤ 0.0001.

Amyloid precursor protein (APP) staining was used to assess axonal damage in the CC of Cup-intoxicated WT and KO mice by quantifying the number of APP^+^ spheroids. As shown in Figure 2, Cup intoxication increased the number of APP-positive spheroids in the medial CC of KO mice in both R1 and R2 compared to WT animals. In R1, the number of spheroids per mm^2^ was 131.21 ± 46.51 in Ctrl mice, 123.19 ± 54.71 in WT Cup mice, and 1568.84 ± 801.75 in KO Cup mice (Figure 2c). In R2, the number of spheroids per mm^2^ was 1470.75 ± 429.80 in Ctrl mice, 1492.75 ± 181.01 in WT Cup animals, and 3311.59 ± 613.90 in KO Cup mice (Figure 2d).

### 2.3. Corpus Callosum Myelination Was Reduced in ASIC1 KO Mice Compared to WT Animals After Cuprizone Intoxication

**Figure 3:**
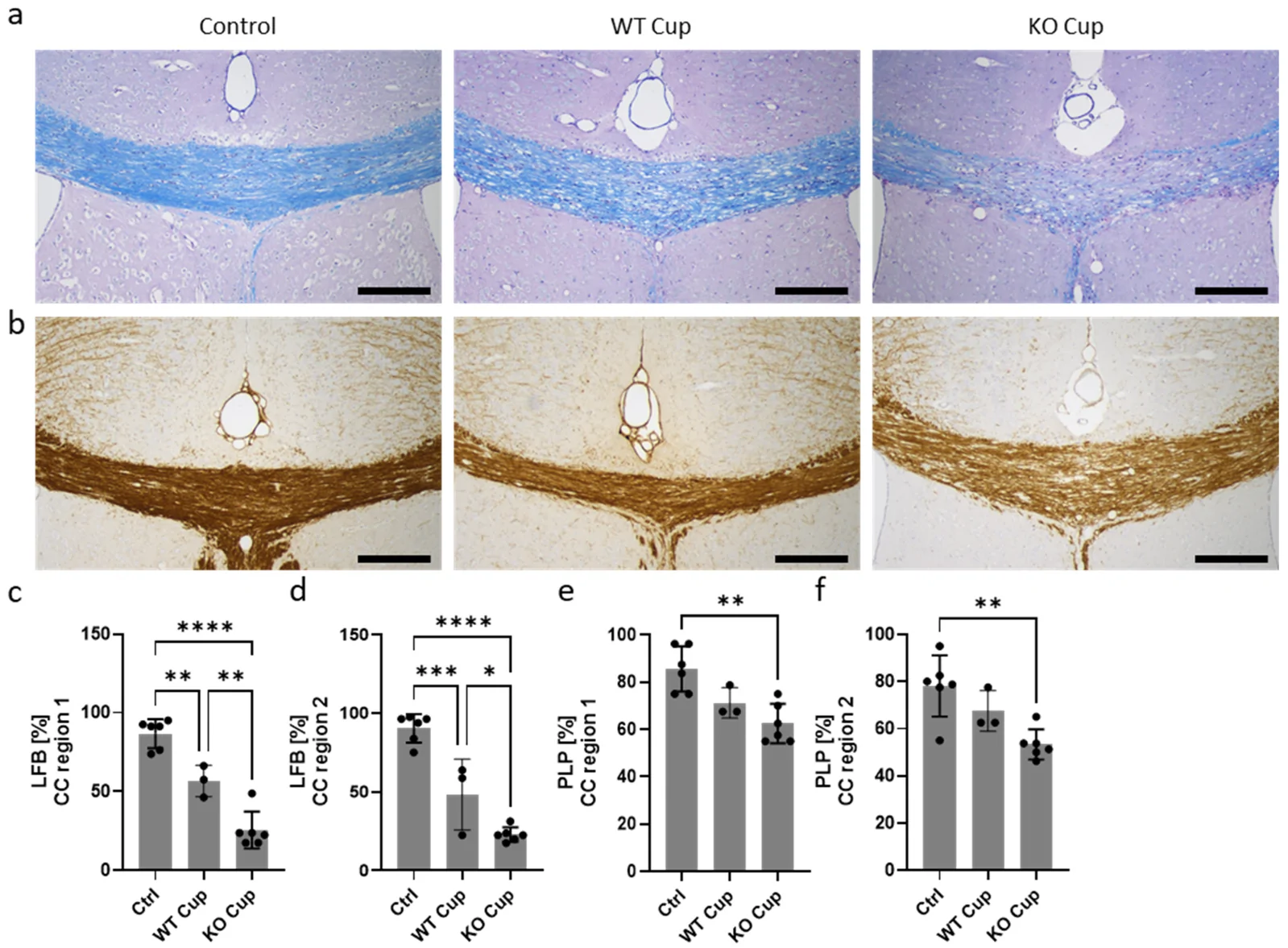
Analysis of myelination in the Corpus Callosum (CC) after 3 weeks of cuprizone intoxication. (**a**) Representative images of LFB staining in the CC of region 1. (**b**) Representative images of PLP staining in the CC of region 1. (**c**,**d**) Staging of LFB^+^ area in the CC of region 1 (**c**) and region 2 (**d**). (**e**,**f**) Staging of PLP^+^ in the CC of region 1 (**e**) and region 2 (**f**). Scale bar 200 µm. *n* = 6 (Ctrl, KO Cup), *n* = 3 (WT Cup). Mean ± SD. ANOVA with Tukey’s multiple comparison. * *p* ≤ 0.05; ** *p* ≤ 0.01; *** *p* ≤ 0.001; **** *p* ≤ 0.0001.

LFB histochemistry and PLP staining were used to assess myelin status in the examined animals, as shown in Figure 3. In R1, LFB revealed a myelination score of 86.67 ± 9.17% in the Ctrl group, 56.67 ± 10.03% in WT Cup mice, and 25.62 ± 11.69% in KO Cup animals (Figure 3c). In R2, 90.42 ± 9.07% of the ROI was positively stained with LFB/PAS in the Ctrl group, compared with 48.33 ± 22.51% in the WT Cup group and 22.92 ± 4.65% in the KO Cup group (Figure 3d). PLP staining was evaluated using a myelination score similarly to LFB histochemistry (Figure 3b). In R1, the myelin score in the CC was 85.62 ± 9.57% in the Ctrl group, 71.25 ± 6.49% in WT Cup mice, and 62.5 ± 8.36% in KO Cup mice (Figure 3e). In R2, 78.12 ± 12.98% of the ROI was positively stained with PLP in the Ctrl group, 67.5 ± 8.66% in WT Cup mice, and 53.33 ± 6.35% in KO Cup mice (Figure 3f). Overall, these findings indicate that Cup intoxication induces myelin loss in the medial CC of mouse brains, and that ASIC1 deficiency further aggravates this effect. PLP staining showed a pattern similar to that of LFB, but the comparison between WT Cup and KO Cup animals did not reach significance.

### 2.4. No Additional Reduction in Oligodendrocyte Number Was Detected in ASIC1 KO Mice After 3 Weeks of Cuprizone Intoxication

**Figure 4:**
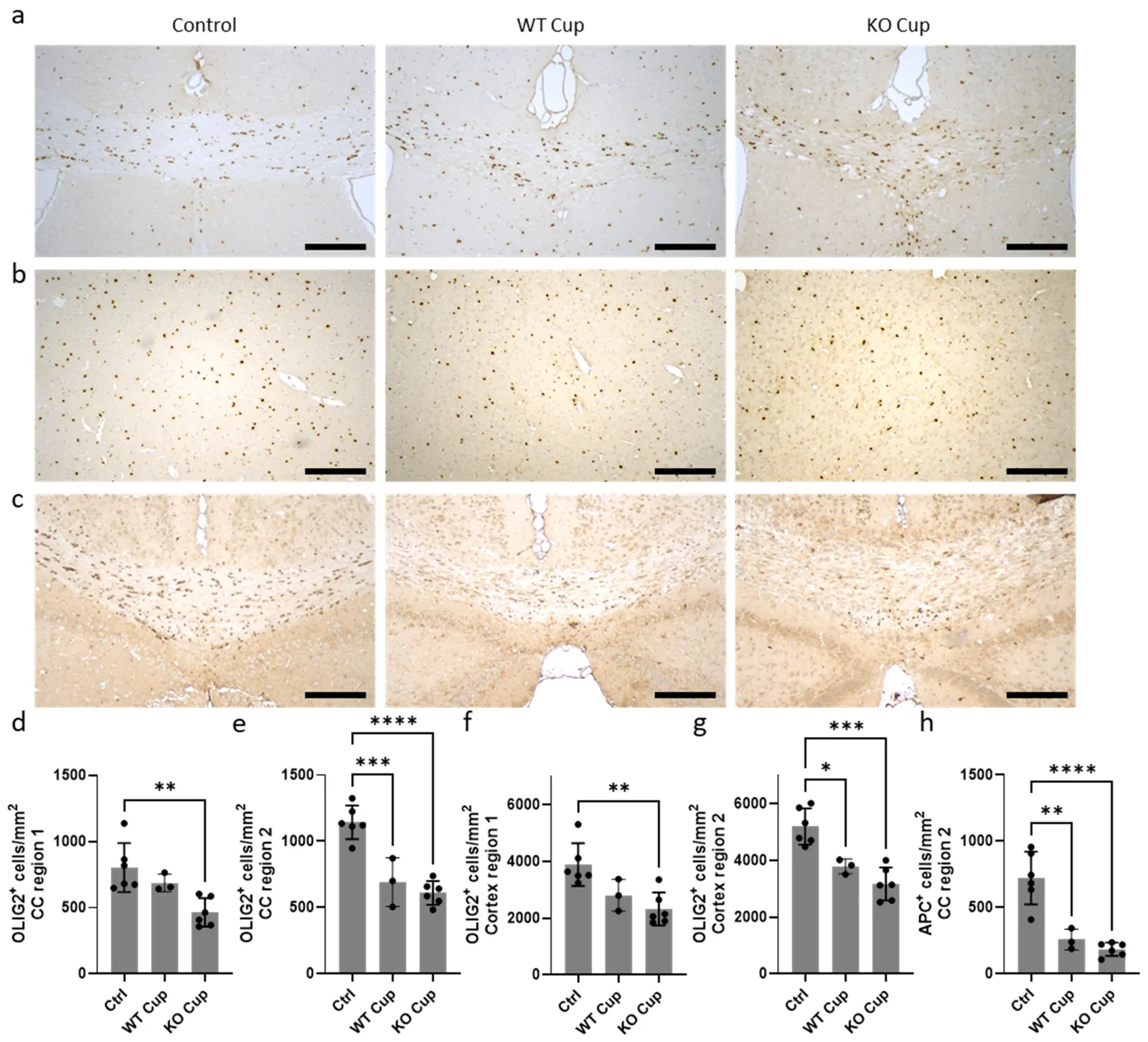
Analysis of oligodendrocytes in the Corpus Callosum (CC) and Cortex after 3 weeks of cuprizone intoxication. (**a**) Representative images of OLIG2 staining in the CC of region 1. (**b**) Representative images of OLIG2 staining in the Cortex of region 1. (**c**) Representative images of APC staining in the CC of region 2. (**d**,**e**) Analysis of OLIG2^+^ cells/mm^2^ in the CC of region 1 (**d**) and region 2 (**e**). (**f**,**g**) Analysis of OLIG2^+^ cells/mm^2^ in the Cortex of region 1 (**f**) and region 2 (**g**). (**h**) Analysis of APC^+^ cells/mm^2^ in the CC of region 2. Scale bar 200 µm. *n* = 6 (Ctrl, KO Cup), *n* = 3 (WT Cup). Mean ± SD. ANOVA with Tukey’s multiple comparison. * *p* ≤ 0.05; ** *p* ≤ 0.01; *** *p* ≤ 0.001; **** *p* ≤ 0.0001.

Because the findings indicated increased axonal damage and demyelination, oligodendrocytes, as the myelinating cells, were evaluated using the markers OLIG2 and APC in both white and gray matter regions (Figure 4) in R1 and R2. In addition, mature APC-positive oligodendrocytes were quantified in R2 CC. In R1, the number of OLIG2^+^ cells per mm^2^ in the CC ROI was 803.69 ± 184.98 in Ctrl mice, 687.08 ± 66.93 in WT Cup animals, and 464.46 ± 108.01 in KO Cup animals (Figure 4d). In R2, the number of OLIG2^+^ cells per mm^2^ was 1143.53 ± 126.12 in the Ctrl group, 692.27 ± 183.74 in WT Cup mice, and 609.42 ± 89.42 in KO Cup mice (Figure 4e). The number of OLIG2^+^ cells in the Cortex was also evaluated (Figure 4b,f,g). In R1, the number of cells was 3885.69 ± 752.87 in Ctrl animals, 2809.70 ± 560.45 in the WT Cup group, and 2326.78 ± 580.78 in KO Cup mice. In R2, the number of cells per mm^2^ was 5197.95 ± 637.06 in the Ctrl group, 3788.31 ± 258.93 in WT Cup animals, and 3173.69 ± 581.27 in KO Cup animals. APC, used as a marker for mature/myelinating oligodendrocytes, was investigated in R2 and showed 721.99 ± 198.67 APC^+^ cells/mm^2^ in Ctrl mice, 256.97 ± 79.13 in WT Cup mice, and 183.39 ± 50.38 in KO Cup mice (Figure 4c,h). Taken together, these results indicate that Cup intoxication decreases the number of OLIG2-positive cells in the medial CC and Cortex in both R1 and R2, as well as the number of APC-positive cells in the CC of R2. The non-mature compartment did not differ between groups, whereas the mature fraction fell in both cuprizone groups without a difference between genotypes (see Figure S4). In these cell populations no additional reduction was detected in ASIC1-KO animals compared to WT.

## 3. Discussion

The present study identifies distinct but potentially converging mechanisms of white matter pathology following cuprizone exposure in ASIC1-deficient animals. As expected, cuprizone exposure led to a reduction in oligodendrocyte number together with decreased LFB and PLP staining, increased APP accumulation, and enhanced IBA1 immunoreactivity, indicating that cuprizone-induced demyelination is accompanied by axonal injury and microglial activation [28,29,30]. Importantly, although ASIC1 deficiency was associated with a more pronounced myelin phenotype, oligodendrocyte loss did not differ between ASIC1-deficient and WT animals. This suggests that the aggravated myelin pathology in the KO is unlikely to be explained simply by enhanced oligodendrocyte death. Instead, our data is rather consistent with the possibility that ASIC1 contributes to myelin maintenance or oligodendrocyte function under cellular stress, while having less influence on oligodendrocyte survival itself, at least at the time point examined. In addition, ASIC1 deficiency does not alter the astroglial response to cuprizone intoxication (see Figure S1). Further, presynaptic VAMP2 immunoreactivity is not altered after 3 weeks of cuprizone intoxication, independently of ASIC1 genotype, which was assessed to investigate whether the aggravated myelin and axonal phenotype of ASIC1 KO animals is accompanied by synaptic changes (see Figure S2).

The selective vulnerability of callosal oligodendrocytes under cuprizone treatment remains mechanistically unresolved. One possible explanation is that copper chelation by cuprizone disrupts mitochondrial function, leading to respiratory chain impairment and subsequent (oxidative) stress (reviewed by [31,32]). Such metabolic disturbances could not only cause oligodendrocyte death, but also compromise oligodendrocyte function and myelin maintenance [32,33]. We hypothesize that ASIC1 channels may contribute to the oligodendrocyte ion homeostasis necessary for myelin maintenance in the case of cuprizone intoxication. This interpretation is consistent with the observed discrepancy between reduced myelin staining in KO on the one hand and relatively unaltered oligodendrocyte numbers comparing WT to KO mice on the other hand.

This interpretation is in line with the broader literature showing that ion channels in oligodendrocyte lineage cells regulate myelination-related processes beyond cell viability. For example, Nav1.2 has been shown to support axon–oligodendrocyte interactions and myelin sheath formation, and selective deletion impaired sheath formation [34]. HCN2 is expressed in mature oligodendrocytes and regulates myelin sheath length as well as expression of key myelin proteins, without primarily altering oligodendrocyte number [35]. Likewise, loss of NCX3 impairs oligodendrocyte differentiation and myelin-related gene expression [36]. More generally, ion channels have been proposed as important regulators of oligodendrocyte lineage progression and remyelination [10], and channel dysfunction has been implicated in demyelinating white matter disorders, including leukodystrophies and inflammatory demyelination [37]. Taken together, these studies support the idea that altered ion channel function can impair myelin integrity even in the absence of major additional cell loss.

Within this framework, ASIC1 may represent another channel contributing to white matter homeostasis. Because ASIC1 is a proton-gated cation channel, it is plausible that it participates in cellular responses to changes in the extracellular microenvironment during cuprizone-induced stress. However, our data do not allow for a direct mechanistic conclusion on how ASIC1 influences myelin integrity in vivo. Untreated ASIC1 KO mice showed no overt demyelination, with myelination scores of 86.67 ± 9.17% (LFB, region 1) and 90.42 ± 9.07% (LFB, region 2). A direct comparison with the untreated wild-type animals of our previous studies is not meaningful, because those animals were assigned the maximal score by definition of the staging scale applied there [28]. Future ultrastructural analyses using electron microscopy should be performed to assess subtle abnormalities, including myelin splitting, altered myelin compaction, or dysmyelination in ASIC1-deficient animals.

The context dependence of ASIC1 function should also be considered. In EAE, ASIC1 knockout reduced clinical deficits and axonal degeneration [38], and ASIC1 inhibition has been suggested to exert neuroprotective and myelin-protective effects in inflammatory demyelination [39]. The same directionality has been reported outside inflammatory demyelination. Blockade or genetic deletion of calcium-permeable ASIC1a is neuroprotective in cerebral ischemia [40,41], amiloride showed evidence of neuroprotection in primary progressive multiple sclerosis [42], and ASIC1 activity has recently been reported to impede myelin sheath regeneration after spinal cord injury [43]. Our findings in the cuprizone model differ from this pattern, which may reflect important differences between inflammatory, ischemic or traumatic injury and toxic–metabolic demyelination. Whereas EAE is dominated by immune-mediated injury and lesion-associated acidosis [39,44,45,46], cuprizone primarily induces oligodendroglial metabolic stress as described before. It is therefore plausible that the consequences of ASIC1 deficiency depend on the pathological context and the cell types that are most affected.

A further implication of our findings is that the worsened myelin phenotype in ASIC1-deficient animals may contribute secondarily to axonal injury. Since intact myelin is essential for axonal support [47], increased APP accumulation may reflect downstream consequences of impaired myelin integrity rather than a primary axonal effect. Similarly, increased IBA1 immunoreactivity may occur in response to myelin disruption and debris accumulation [48]. While this sequence remains speculative in the absence of direct mechanistic markers in vivo, it is consistent with the overall pattern observed here.

Several limitations of the current study should be considered. First, all read-outs were obtained at a single time point, after 3 weeks of cuprizone intoxication. Cuprizone-induced de- and remyelination is a highly dynamic process involving oligodendrocyte death, precursor recruitment and differentiation, and a single time point cannot resolve these phases or distinguish a delay from a true difference in extent. Second, group sizes were small. The study is therefore powered to detect only large effects, and the absence of a genotype difference in oligodendrocyte numbers should be interpreted as no detectable difference rather than as evidence of no difference. Third, the untreated control group consisted of ASIC1-deficient animals, so that comparisons between untreated controls and cuprizone-fed WT animals combine a genotype and a treatment difference, and no untreated WT reference was available within this dataset. Fourth, the structural properties of myelin were not assessed at the ultrastructural level, leaving the precise nature of the myelin defects in ASIC1-deficient animals unresolved. Fifth, the lack of a cell type-specific knockout model limits conclusions about an oligodendrocyte-autonomous role of ASIC1, since ASIC1 is also expressed in neurons, astrocytes and microglia. Finally, no remyelination arm was included, so the role of ASIC1 in myelin repair remains open.

Future studies should therefore focus on detailed ultrastructural analyses to define the specific myelin abnormalities associated with ASIC1 deficiency. The generation of an oligodendrocyte-specific ASIC1 knockout model would be instrumental in determining whether the observed phenotype arises from cell-intrinsic mechanisms.

## 4. Materials and Methods

### 4.1. Mice, Tissue Preparation and Experimental Design

All experiments were approved by the Review Board for the Care of Animal Subjects of the district government (LANUV, Recklinghausen, North Rhine-Westphalia, Germany; 84-02.04.2017.A117; approval date 7 June 2017) and performed according to the international guidelines on the use of laboratory mice. Wild-type C57BL/6J mice (WT) were acquired from Janvier Labs (Le Genest-Saint-Isle, France). The C57BL/6 ASIC1-deficient animals (KO) were kindly provided by John A. Wemmie, Michael J Welsh and colleagues [49]. C57BL/6 ASIC1-deficient animals were used as control animals (Ctrl). Because the aim of this study was to determine whether ASIC1 deficiency modifies cuprizone-induced pathology, the untreated control group (Ctrl) consisted of ASIC1-deficient animals fed standard chow and is therefore genotype-matched to the cuprizone-treated knockout group (KO Cup). An untreated WT group was not included. The Corpus Callosum phenotype of untreated C57BL/6 mice has been characterized in detail for all read-outs used here, including in our own previous studies employing the same protocols, regions and scoring procedures [28,29]; in accordance with the 3R principles, we considered it unjustified to include a fourth group that would largely reproduce published values. As a consequence, comparisons between the Ctrl and WT Cup groups combine a genotype and a treatment difference, and no untreated wild-type reference is available within this dataset; this is addressed in the Discussion. A total of 15 animals was used for this study: Ctrl *n* = 6, WT Cup *n* = 3, and KO Cup *n* = 6. No a priori power calculation was performed; group sizes were based on previous studies using the cuprizone model in our laboratory. Criteria for exclusion of animals were defined a priori via humane endpoints (e.g., rapid weight loss ≥ 20%, severe dehydration, wounds) and monitored daily. Data point exclusion was defined for histological analysis as follows: damaged sections, folded tissue, and staining artifacts. No animals were excluded from the study and no data points were excluded from the analyses shown in Figure 1, Figure 2, Figure 3, Figure 4 and Figure S1. For the VAMP2 immunofluorescence (Figure S2), some sections had to be excluded for the aforementioned reasons; the number of animals analyzed per group and region is given in the legend of Figure S2. All animals were housed under standard laboratory conditions according to the Federation of European Laboratory Animal Science Association’s recommendations and further bred in house without any pathological phenotype. KO animals were randomly allocated to groups at cage level. Food and water were accessible ad libitum and a 12 h light/dark cycle was applied. The study protocol was not pre-registered. To investigate the possible role of ASIC1 deficiency on the experimental demyelination model, 8-week-old male/female animals were fed with 0.25% (*w*/*w*) cuprizone (Cup, bis-cyclohexanone oxaldihydrazone, Sigma-Aldrich Inc., Darmstadt, Germany) containing standard powder rodent chow or standard chow as control for 3 weeks. The chow was freshly prepared every day and after the feeding period, animals were deeply anesthetized with ketamine/xylazine (100 and 10 mg/kg i.p. with 100 μL/10 g body weight). Tissue was prepared for histology by transcardial perfusion with ice-cold phosphate-buffered saline (PBS), followed by 3.7% paraformaldehyde (pH 7.4; Roth, Karlsruhe, Germany, CP10.2). Whole brains were collected and incubated in the same fixative overnight for postfixation. For tissue embedding, brains were rinsed for 4 h in running tab water. Afterwards, brains were transferred to a 70% ethanol solution for overnight storage at 4 °C. The next day, the tissues were passed through an ascending alcohol series (70%/96%/100% ethanol solution). After that, tissues were incubated in xylol, embedded in 61 °C paraffin, and stored at 4 °C. Tissue was cut with a microtome (Leica Biosystems, RM2255, Nussloch, Germany) into 5 μm thick coronal sections. Investigated brain regions included bregma levels 0.14 mm (region 1, R1) and −0.94 mm (region 2, R2) based on the mouse brain atlas from Paxinos and Franklin [50]. The experimental unit was the individual animal. Two slides were investigated and averaged to yield a single value per staining, region, and animal. To minimize potential confounders, measurement order was randomized and all measurements were conducted blinded for treatment groups and genotype.

### 4.2. Immunohistochemistry

Brain sections were deparaffinized, rehydrated and heat-unmasked in either citrate (pH 6.0) or tris/ethylenediaminetetraacetic acid (Tris-EDTA) buffer (pH 9.0) for antigen retrieval (see Table 1). After cooling down for 10 min, slides were washed with 1× PBS, followed by 1 h incubation with a blocking solution (1% normal horse or normal goat serum in PBS) to avoid unspecific antibody binding. Then, sections were incubated overnight at 4 °C with the respective primary antibody diluted in blocking solution (see Table 1). The next day, a 30 min peroxidase block with 0.3% H_2_O_2_ in PBS was applied. Slices were incubated for 1 h with the appropriate biotinylated secondary antibody (diluted 1:50 in blocking solution; see Table 1) at room temperature, followed by the Avidin/HRP-Biotin Coupling (VECTASTAIN Elite ABC Kit Standard; Vector Laboratories, Newark, CA, USA) for 30 min. For visualization by 3′3-diaminobenzidine, the working solution (1:50, pH 7.5, DAKO, Hamburg, Germany) was applied for 10 min. Where appropriate, slides were counterstained with Mayer’s Hematoxylin (45 s) to visualize cell nuclei. After dehydration in an ascending alcohol series and xylol incubation for 20 min, the sections were mounted in DePeX (Serva, Heidelberg, Germany). For VAMP2 immunofluorescence, sections were deparaffinized and rehydrated through a descending alcohol series, and antigen retrieval was performed in citrate (pH 6.0) buffer for 20 min. After cooling for 10 min and washing in 1× PBS, sections were blocked for 1 h in 0.2% gelatine in PBS and incubated overnight at 4 °C with anti-VAMP2/synaptobrevin 2 antibody diluted 1:500 in blocking solution. The following day, sections were washed and incubated for 1 h at room temperature with anti-mouse Alexa Fluor 546 diluted 1:1000, counterstained with DAPI and mounted in Immu-Mount. Luxol fast blue (LFB)/periodic acid-Schiff staining (PAS) was performed by deparaffinization and rehydration through an ascending alcohol series. Afterwards, the slices were incubated with LFB solution (0.1 g LFB (Roth, Karlsruhe, Germany) + 500 mL acetic acid (Roth, Karlsruhe, Germany) and 100 mL ethanol) overnight at 56 °C. The following day, excess LFB solution was rinsed off with 95% ethanol, and a 0.05% lithium carbonate solution (Merck Millipore, Darmstadt, Germany) was applied for 15 s. Next, the sections were soaked in a 70% ethanol solution for 10 s. For PAS staining, slices were oxidized for 5 min by 0.5% periodic acid solution, washed in distilled water and incubated for 15 min in Schiff reagent (Merck Millipore, Darmstadt, Germany). The counterstaining was performed using hematoxylin for 1 min after rinsing the sections in warm water. Finally, after another washing step, slides were dehydrated and embedded.

### 4.3. Analyses and Statistics

Immunohistochemical results for IBA1- and GFAP-positive area, as well as APP-, OLIG2-, and APC-positive cell numbers per mm^2^, were analyzed using ImageJ, version 1.54 [51]. Analyses in the Corpus Callosum were performed within a defined region of interest (ROI), which is illustrated in Figure S3. The ROI comprised the medial CC and extended across the full width of the captured image; its dorsal and ventral boundaries were formed by the borders between white and gray matter. The identical ROI was used for all stainings and for both regions R1 and R2. Separate regions of interest were used for the cortical OLIG2 analysis (whole image; Figure 4b,f,g) and for the VAMP2 analysis (Figure S2), as specified in the respective figure legend. LFB/PAS and PLP staining were assessed within the same ROI, but by visual staging rather than densitometry: at least two investigators blinded to genotype and treatment independently assigned a myelination score from 0% = complete demyelination to 100% = normal myelination, and the scores were averaged. Images of VAMP2 staining were acquired on a Keyence BZ X810 (Osaka, Japan) with 40× objective using identical exposure settings for all sections within the staining batch. They were analyzed using ImageJ to evaluate mean fluorescence intensity. Mean fluorescence intensity of the CC was subtracted as background from every other ROI in the same slice. The results in this study are given as mean ± standard deviation (SD). The tests used to evaluate the significance of mean differences among the groups are given in the figure legends. Prior to data analysis, normal distribution and homogeneity of variance were assessed using the Shapiro–Wilk test and the Brown–Forsythe test; given the small group sizes, these tests have limited power. Data were evaluated using one-way analysis of variance (ANOVA) with Tukey’s HSD post hoc test. The data were analyzed using GraphPad Prism 10 (GraphPad Software Inc., San Diego, CA, USA) and *p* < 0.05 was considered significant.

## Figures and Tables

**Table 1:** Antibodies used for immunohistochemistry.

Antibody	Species/Clonality	Concentration	Antigen Retrieval	Company
IBA1	Rabbit polyclonal	1:10,000	Tris-EDTA (10 min)	FUJIFILM WAKO Chemicals, Osaka, Japan
GFAP	Goat polyclonal	1:1000	Tris-EDTA (20 min)	Santa Cruz, Dallas, TX, USA
OLIG2	Mouse monoclonal	1:2000	Citrate (10 min)	Merck Millipore, Burlington, MA, USA
APC (Ab-7), clone CC1	Mouse monoclonal	1:250	Tris-EDTA (20 min)	Merck Millipore, Burlington, MA, USA
APP	Mouse monoclonal	1:5000	Tris-EDTA (10 min)	Merck Millipore, Burlington, MA, USA
PLP	Rabbit polyclonal	1:1500	Tris-EDTA (10 min)	Abcam, Cambridge, UK
VAMP2	Mouse monoclonal	1:500	Citrate (20 min)	Synaptic Systems, Göttingen, Germany
Biotinylated anti-mouse IgG (H + L)	Goat	1:500	/	Vector laboratories, Inc., Newark, CA, USA
Biotinylated anti-rabbit IgG (H + L)	Goat	1:500	/	Vector laboratories, Inc., Newark, CA, USA
Biotinylated anti-goat IgG (H + L)	Horse	1:500	/	Vector laboratories, Inc., Newark, CA, USA
Alexa Fluor 546 anti-mouse	Goat	1:1000	/	Invitrogen, Thermo Fisher Scientific, Waltham, MA, USA

## Data Availability

The original contributions presented in this study are included in the article/Supplementary Materials. Further inquiries can be directed to the corresponding author.

## Supplementary Materials

The following supporting information can be downloaded at https://www.mdpi.com/article/10.3390/ijms27219758/s1.

## Abbreviations

The following abbreviations are used in this manuscript:

ANOVA	Analysis of variance
APC	Anti-adenomatous polyposis coli
APP	Amyloid precursor protein
ASIC1	Acid-sensing ion channel subtype 1
CC	Corpus callosum
CNS	Central nervous system
Ctrl	Control
Cup	Cuprizone
DAPI	4′,6-diamidino-2-phenylindole
ENaC/DEG	Epithelial sodium channel/degenerin
GFAP	Glial fibrillary acidic protein
IBA1	Ionized calcium-binding adaptor molecule 1
KO	Knockout
LFB/PAS	Luxol fast blue/periodic acid-Schiff
MS	Multiple sclerosis
OLIG2	Oligodendrocyte transcription factor 2
PBS	Phosphate-buffered saline
PLP	Proteolipid protein
R1	Region 1
R2	Region 2
ROI	Region of interest
ROS	Reactive oxygen species
SD	Standard deviation
Tris-EDTA	Tris/ethylenediaminetetraacetic acid
VAMP2	Vesicle-associated membrane protein 2 (synaptobrevin 2)
WT	Wild-type
